# The London School of Clinical Medicine

**Published:** 1906-02-24

**Authors:** 


### THE LONDON SCHOOL OF CLINICAL MEDICINE.

An interesting event took place on Thursday last at the Seamen's Hospital, Greenwich, when some 150 medical practitioners, the majority of whom were members of the Norwood Division of the British Medical Association, paid a visit to the hospital and inspected the arrangements which have been made for teaching purposes in connection with the new London School of Clinical Medicine, lately established in connection with that hospital. In the course of an address in which, on behalf of his colleagues on the medical staff, he welcomed the visitors, Sir Dyce Duckworth referred to the notorious fact that until recently London had not kept pace with many continental centres in supplying facilities for post-graduation medical study. The consequence of this neglect was that many members of the profession who wished to freshen their knowledge of general medicine, or to acquire proficiency in any special branch of it, had been obliged in the past to turn their backs on London and go abroad. Now, however, there were several centres in London where good arrangements were made for men to carry on their studies and complete them; and among these he included the London School of Clinical Medicine at Greenwich, where an excellent equipment had been established, with the kind co-operation of the governing body of the Seamen's Hospital. Dr. Guthrie Rankin, who also spoke, remarked upon the variety of clinical material which this hospital contained, and which was interesting from every point of view. Although their cases were practically limited to men, in order to make the school as useful as possible, arrangements had been made to affiliate with themselves the Royal Waterloo Hospital for Children and Women, the Royal Bethlehem Hospital, and the York Road Lying-in Hospital; so that every department of work that could not be undertaken at the Seamen's Hospital could be carried out at those institutions. He said that those who had organised the London School of Clinical Medicine looked forward to the day when, if they were supported by the public and the governing body, work might be done in that school of the same type as that with which they were all familiar as coming from the Johns Hopkins University in America.

After the meeting the visitors inspected the school and hospital, and demonstrations of numerous interesting medical and surgical cases were made by members of the honorary staff.

